# Seasonal Variation of Midgut Bacterial Diversity in *Culex quinquefasciatus* Populations in Haikou City, Hainan Province, China

**DOI:** 10.3390/biology11081166

**Published:** 2022-08-03

**Authors:** Penghui Suo, Kaixuan Wang, Hongxiao Yu, Xiuhao Fu, Liping An, Biswajit Bhowmick, Jiachao Zhang, Qian Han

**Affiliations:** 1 Laboratory of Tropical Veterinary Medicine and Vector Biology, School of Life Sciences, Hainan University, Haikou 570228, China; 19071005210001@hainanu.edu.cn (P.S.); 20086000210041@hainanu.edu.cn (K.W.); yhxhainanu@163.com (H.Y.); fxhhainan8688@163.com (X.F.); 19071007210001@hainanu.edu.cn (L.A.); biswajit.bhowmick@hotmail.com (B.B.); 2 One Health Institute, Hainan University, Haikou 570228, China; 3 College of Food Science and Engineering, Hainan University, Haikou 570228, China; zhjch321123@163.com

**Keywords:** *Culex quinquefasciatus*, 16S rDNA, diversity of midgut bacteria, environmental factors

## Abstract

**Simple Summary:**

Mosquito midgut microbiota has become an interesting field in mosquito vector biology, as it has been shown to form an integral part of the mosquito life history. But less is known about seasonal variation of midgut bacterial diversity of *Culex quinquefasciatus.* Our results illustrate that the Bacteroidetes (Bacterial Phyla) communities have been well observed in autumn and winter seasons, suggesting that this might participate in the nutritional supply of adult mosquitoes when temperatures drop. This discovery provides a new perspective for the control of *Cx. quinquefasciatus* to reduce the transmission of diseases. There is much sufficiently practical significance to reduce the density of *Cx. quinquefasciatus* in autumn and winter when their activities are weakened, which is of absolute benefit to human beings and the natural environment.

**Abstract:**

*Culex quinquefasciatus*, one of the most significant mosquito vectors in the world, is widespread in most parts of southern China. A variety of diseases including Bancroft’s filariasis, West Nile disease, and St. Louis encephalitis could be transmitted by the vector. Mosquitoes have been shown to host diverse bacterial communities that vary depending on environmental factors such as temperature and rainfall. In this work, 16S rDNA sequencing was used to analyze the seasonal variation of midgut bacterial diversity of *Cx. Quinquefasciatus* in Haikou City, Hainan Province, China. Proteobacteria was the dominant phylum, accounting for 79.7% (autumn), 73% (winter), 80.4% (spring), and 84.5% (summer). The abundance of Bacteroidetes in autumn and winter was higher than in others. Interestingly, *Epsilonbacteraeota,* which only exists in autumn and winter, was discovered accidentally in the midgut. We speculated that this might participate in the nutritional supply of adult mosquitoes when temperatures drop. *Wolbachia* is the most abundant in autumn, accounting for 31.6% of bacteria. The content of *Pantoea* was highest in the summer group, which might be related to the enhancement of the ability of mosquitoes as temperatures increased. *Pseudomonas* is carried out as the highest level in winter. On the contrary, in spring and summer, the genus in highest abundance is *Enterobacter*. *Acinetobacter* enriches in the spring when it turns from cold to hot. By studying the diversity of midgut bacteria of *Cx. quinquefasciatus*, we can further understand the co-evolution of mosquitoes and their symbiotic microbes. This is necessary to discuss the seasonal variation of microorganisms and ultimately provide a new perspective for the control of *Cx. quinquefasciatus* to reduce the spread of the diseases which have notably vital practical significance for the effective prevention of *Cx. quinquefasciatus*.

## 1. Introduction

In the ecosystem, microbes play a vital role in various physiological aspects in the life of other organisms including animals, plants, and even human beings. Thereby, microorganisms are called one of the most significant components of the ecosystem by scientists [1]. Additionally, various insects host resident microorganisms that are able to influence many insects’ key functions. Among microbes, tens of thousands of microorganisms, such as bacteria, fungi, viruses, and archaeobacteria, exist in various insects [2]. Ordinarily, the surface skin, internal genitalia, gut, and other parts of insects in particular are parasitized by microbes. Existing studies have suggested that, directly or indirectly, the physiological activities of insects were regulated by microbial communities in many aspects, such as growth and development, reproduction of offspring, nutrition and metabolism, neural and immune regulation, etc. [2]. Interestingly, the number of symbiotic microorganisms were accounted for the largest community of insect cells. Multiple biological response mechanisms in insects could be regulated by their microbial community, which is composed of microbial cells and genes [3,4,5,6].

Previous research has shown that symbiotic microbes enabled insects to survive in poor nutritional conditions by supplying necessary nutrients and assisting digestion [7]. In the gut of the tsetse flies under a lack of natural food, *Wigglesworthia glossinidia* produce vitamin B and some symbiotic microbes synthesize other nutrients [8]. Additionally, it was shown that *Buchnera* in aphids could generate a variety of essential amino acids to make up for the nutritionally poor food that was lacking some amino acids and vitamins [9]. Meanwhile, the antibiotics or toxins secreted by symbiotic microbes might also prevent pathogens from invading the host: in female digger wasps, *Streptomyces albus* was used to resist the infection of fungi [10]. Practically, in *Dendroctonus frontalis* and *Tetramorium*, antimicrobial substances secreted by symbiotic microbiota could inhibit not only harmful fungi but also other reciprocal fungi [11,12]. However, the role of symbiotic microbiota is not always positive and can affect hosts negatively. For example, the armyworm colonized by *Wolbachia* was significantly more sensitive to nuclear polyhedrosis viruses. In the case of *Drosophila melanogaster*, symbiotic *Wolbachia* had no effect on host’s resistance against pathogenic bacteria. On the contrary, some bacteria such as *Spiroplasma* would enhance the sensitivity to certain pathogenic bacteria. There are also many factors, such as host species, genetic information, life cycle, environmental conditions, and the type of symbiotic bacteria, that may affect the relationship between symbiotic microbes and pathogens. The specific mechanism of action of these factors is worthy of more in-depth study. Afterward, studies on the gut microbes which enhanced host detoxification [13,14,15,16,17,18] and produced insecticide resistance have also been repeatedly reported [19,20]. Trichlorfon, used for the pest control of *Bactrocera dorsalis,* is an organophosphorus insecticide with low toxicity and high insecticidal efficiency. Researchers have found that trichlorfon can be degraded by *Citrobacter freundi-BD* (CF-BD) which can effectively heighten the resistance to trichlorfon insecticide. Recently, the environmental adaptability of insects affected by their endogenous microbes has also been researched [21]; the existence of symbiotic microorganisms could improve the adaptability of aphids to high temperature and thus the rate of infection of other symbiotic microbes in aphids boosted considerably. Likewise, it was speculated by scientists that the protein molecules of the host may be protected from degradation by this mechanism due to the increasing gene expression of the bacterial molecular chaperone GroEL [22]. In the infection experiment, the researchers also confirmed that the fecundity of pea aphids (*Acyrthosiphon pisum*) infected by *Regiella* definitively increased [23], but this special performance only appeared in specific strains of pea aphids rather than all strains, suggesting that the interaction between microbiota and hosts was special firmly from another perspective [24]. Even more surprising is the fact that the interaction between symbiotic microbes has also been revealed. The genome of *T. princeps* in the evolution of *Planococcus citri* has been degraded. Simultaneously, some genes that are related to translation proteins have been directly lost. Nevertheless, studies have shown that some related proteins can be produced in the presence of *M. endobia*, which can be used by *T. princeps* to restore the protein translation pathway due to gene loss. Both also showed a typical dependency correlation.

Currently, vector-borne diseases are one of the major problems facing human beings, especially those transmitted by mosquitoes [25]. *Cx. quinquefasciatus*, one of the most significant mosquito vectors in the world, is widespread in most parts of southern China with a wide range of distribution. A variety of diseases could be spread, including bancroftian filariasis, West Nile encephalitis, St. Louis encephalitis [26,27,28], etc. There is no doubt that these studies were exceedingly necessary for mosquito-borne diseases, but there was a lack of depth exploration of the processes affecting mosquito-pathogen interactions. Given this, the focus of the latest research is shifting to the mechanism of vector organisms being infected by pathogens and the development of new control strategies. With the continuous advancement of bioscience, it was revealed that the microbial community has become a key factor driving many physiological aspects of the host [29], including the host’s development, nutrition, survival, and the ability of pathogens to spread through insect vectors. Nowadays, more attention has been attracted to the connection between drug resistance and symbiotic microbes of mosquitoes. Compared with previous research on mosquito resistance, most of them focused on mosquito resistance genes. The resistance of *Anopheles* to pesticides could be increased by gut microbes promoting metabolic activity to organophosphorus insecticides [30]. *Wolbachia* was highly enriched in deltamethrin-resistant *Culex pipiens pallens*, indicating that *Wolbachia* was related to the resistance to deltamethrin [31]. Afterward, *Klebsiella pneumoniae* accumulated in fenitrothion-resistant strains of *Anopheles* [32]. These results all reasonably opened out that there was a certain correlation between the midgut microbes and drug resistance of mosquitoes.

Regrettably, most of the understanding regarding the microbes in mosquitoes is based on the species in the laboratory, and there was a lack of verification of data collected in the wild [33]. Therefore, the time and cost of epidemiological research could be extremely decreased by due to wild mosquito species collection, and the dynamics of midgut microbes of mosquito can be effectively captured to analyze the specificity of general species [34]. Apart from this, the current measures for the prevention and control of mosquito vectors were mainly chemical control. However, due to the long-term abuse of chemical pesticides which also polluted the environment and was not conducive to human health, the issue of drug resistance of mosquitoes has become increasingly prominent [35]. Long-lasting, safer, and environment-friendly biological control measures are becoming peculiarly popular among researchers. It can be seen that mosquito-related microbes, which can respond quickly to changing environmental conditions and promote the adaptation and evolution of the host, were the most neglected but more dynamic component of mosquitoes. Furthermore, the resolution of the individual and inter-individual variation of microbes of mosquitos, which was the fertile soil for mosquito innovation, was of great significance for the prevention of disease vectors. Thus, the exploration of the variability of the microbes of mosquitoes is currently becoming an extremely notable method to progress new strategies of vector control based on microorganisms. Understanding the role of microbes in host evolution, adaptation, and physiology including pathogen acquisition, resistance, and transmission, is the key to efficient and sustainable biological prevention [36]. In this work, midgut microbes in wild samples of *Cx. quinquefasciatus* were selected for 16S rDNA sequencing. The population diversity was analyzed in order to clarify the relationship between *Cx. quinquefasciatus* and its gut microorganisms. Our work is going to contribute to the biological control measures of pollution-free insect control based on gut bacteria to fully explore microbial reagent and to improve the utilization of related biological resources for the protection of the ecological environment [37,38,39].

## 2. Materials and Methods

### 2.1. Mosquito Collection

The *Cx. quinquefasciatus* was collected from September 2020 to September 2021 in the living areas of urban residents in Haikou City, Hainan Province, China. Monitoring was done 3 times a month with an interval of 10 days (Merge standard: after each monitoring, the samples were collected and placed separately as a repetition of the experiment in each season). The adult mosquitoes were captured by the trapping method by humans, double-layered netting method, and CO_2_ ultraviolet lamp trapping method. The monitoring started 1 h after sunset and lasted for 10 h each time, and the collected mosquitoes were reared at 26 ± 1 °C, 75 ± 5% of relative humidity in a cage (125 cm × 105 cm × 95 cm). The selected sites and capture method remained unchanged throughout the whole sampling process (Supplementary Table S1). After the completion of the monitoring, the collected samples were counted and identified by morphology and molecular biology and were subsequently preserved and dissected in four seasons for later experiments (Supplementary Table S2).

### 2.2. 16S rDNA Sample Preparation of Mosquito Midgut

The adult mosquitoes of *Cx. quinquefasciatus* collected in the field were classified, and the adult female mosquitoes were taken for this study. It is worth noticing that the adult female mosquitoes were usually starved for 24 h to avoid the influence of passing bacteria in food in the process of extracting symbiotic microbes [40]. Under aseptic conditions, female mosquitoes were disinfected with 75% ethanol for 90 s on the surface of *Cx. quinquefasciatus* and then rinsed with sterile water three times. Subsequently, the midgut of each female mosquito dissected under a microscope was gently removed to a sterile 1.5 mL centrifuge tube containing 100 μL phosphate-buffered saline (PBS). Each tube contained 40–60 midgut tissues, and the experiment was repeated in triplicate. The microbial genomic DNA of the 16S rDNA samples were extracted with the HiPure Stool DNA Kits (Guangzhou, China). While collecting the midgut of female mosquitoes, tissues other than the midgut were quickly and thoroughly ground with liquid nitrogen for 2 min and collected into a sterile 1.5 mL centrifuge tube containing PBS. Successively, the genomic DNA of the tissues of female mosquitoes was extracted with Ezup Column Animal Genomic DNA Purification Kit (Shanghai, China) for molecular identification of mosquito species. After that, Qubit and NanoDrop were used to detect the concentration and the purity of DNA. Furthermore, PCR and agarose gel electrophoresis were used to detect Genomic DNA integrity. Then, the samples stored in dry ice were sent to Genedenovo Biotechnology Co. (Guangzhou, China) for genome quality inspection, library construction, sequencing, and biological information analysis.

### 2.3. Analysis Process

ITS is widely used in mosquito species identification, because mature ribosomes are not contained in the ITS region, and thus have less evolutionary pressure and a faster evolution rate. In this study, we used the ITS2 region (F: GGGGTAGTCACACATTATTTGAGG, R: GAACTGCAGGACACATGAACACCG) as a molecular marker for species identification [41]. 

V3–V4 regions of bacteria were selected for amplification (341F: CCTACGGGNGGCWGCAG, 806R: GGACTACHVGGGTATCTAAT). After the raw reads were obtained by sequencing, the software of FASTP and FLASH were used to filter low-quality reads and to splice the double-ended reads into tags. After filtering the tag, the data obtained at this time was called Clean Tag. Subsequently, the UPARSE9.2.64 pipeline was used to cluster valid tags into an operational taxon (OTU) with ≥97% similarity based on the Clean Tag. We removed the chimera tag detected in the clustering comparison process by USEARCH software, and the Effective Tag was used in OTU abundance statistics. In the process of constructing OTUs, the algorithm of Naïve Bayesian assignment of RDP Classifier was used to annotate species within the database with the confidence threshold as 0.8:1. After getting the species annotation information of each OTU, we utilized KRONA to visually display the results. The taxonomic ranks were domain, phylum, class, order, family, genus, and species. In addition, Alpha-diversity and Beta-diversity were calculated with QIIME. Venn analysis was performed using the VennDiagram package. Additionally, Heatmaps were generated using Omicsmart, a dynamic real-time interactive online platform for data analysis (Available online: http://www.omicsmart.com (accessed on 5 November 2021)).

### 2.4. Bioinformatic and Sequence Analysis

In this study, through the results of the molecular identification of samples, the mosquitos were identified as *Cx. quinquefasciatus*. A total of 6,610,700 pairs of Raw Tags were obtained from 52 midgut pooled samples of *Cx. quinquefasciatus* by sequencing. After merging, a total of 6,584,002 Clean Tags were generated, with an average of 126,615 ± 5217 Clean Tags and 113,584 ± 6116 Effective Tags per sample. The Effective Ratio was over 87.75%. In Effective Tags, maximum and minimum lengths were 472 ± 2 tags and 211 ± 1 tags, respectively. The mean value of N50, a part of assessing assembly quality, was above 463 tags, indicating that the sample gene assembly was of high integrity and good quality which ensured the reliability of the subsequent analysis (Supplementary Table S3).

**Figure 1:**
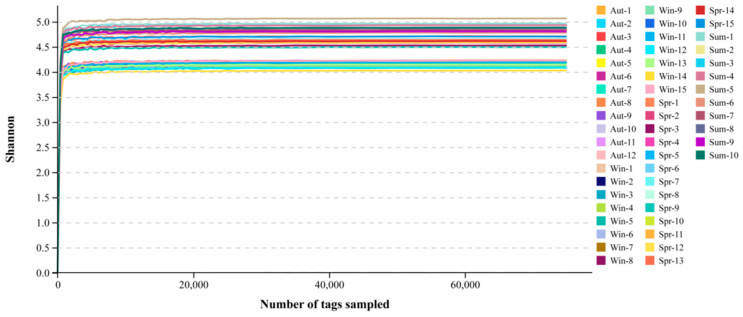
Dilution curve of shannon index. The abscissa is the number of fragments, the ordinate is the shannon index. Different samples were represented by different colors.

The dilution curve, which is used to evaluate whether the amount of sequencing data was sufficient to cover all the labeled sequences contained in the sample, can indirectly reflect the abundance of species in the sample. As can be seen from the species accumulation curve (Figure 1), the slope of the curve rose sharply with increasing sequencing depth, indicating that a large number of microbial diversities were effectively captured at this time. The growth rate of the curve slowed down when the number of sequences was around 12,000 tags. While the sequences were above 16,000 tags, the dilution curve had a plateau at this moment, which illustrated that the sequencing depth of the sample was large enough to cover the vast majority of microbial information in the sample. If the amount of sequencing data was increased, no more OTU can be found overwhelmingly, which was suitable for the evaluation and analysis of microorganisms.

**Figure 2:**
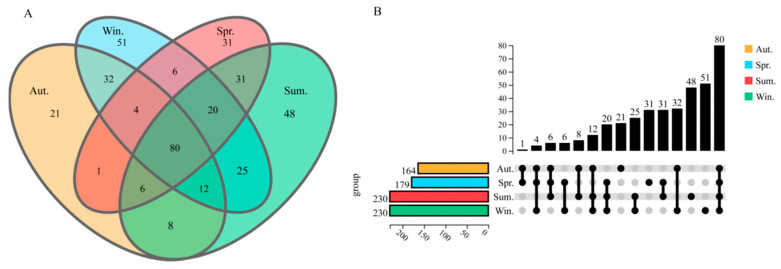
Venn diagram and upgrade plot. In the Venn diagram, the overlapping part represents the number of homologous communities, and different color groups represent sampling samples in different seasons (**A**). In the upgrade plot, the number of shared and unique bacteria genera among the microbiota in the Autumn (orange), Winter (green), Spring (blue), and Summer (red) (**B**).

All samples were clustered at an operating level of 97%, with an average of 398 ± 63 OTUs. Figure 2 was obtained by comparing the OTUs between the samples, and a total of 376 bacteria were annotated by sequencing at the genus level. Among them, 80 strains (21.27%) were homologous in four seasons. There were 21, 51, 31, and 48 species unique to the midguts of the groups of autumn, winter, spring, and summer, accounting for 5.6%, 13.6%, 8.2%, and 12.8% of the total species, respectively. Furthermore, we found that 1, 8, 32, 31, 6, and 25 species of bacteria were discovered in the groups of autumn and spring, autumn and summer, autumn and winter, spring and summer, spring and winter, and summer and winter, respectively.

### 2.5. Statistical Analysis

Differences between groups were analyzed using STAMP. The significance of differences between three or more groups was analyzed by one-way analysis of variance (ANOVA) and Tukey’s multiple comparisons tests. The standard *t*-test and Wilcoxon test were performed to analyze data between two groups, *p*-values < 0.05 were considered statistically significant: the smaller the *p*-value, the more significant the difference.

## 3. Results

### 3.1. The Microbiota Composition of Midgut in Cx. quinquefasciatus

**Figure 3:**
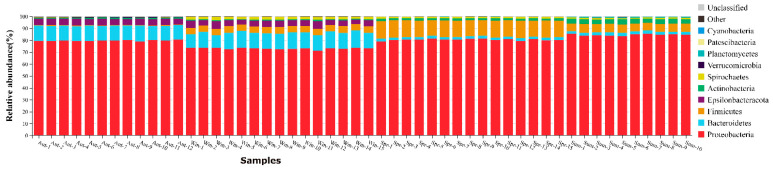
The taxonomic composition of the dominant bacteria of *Cx. quinquefasciatus*, the abscissa and ordinate are samples and the relative abundance respectively. Different colors represent different bacteria (at the phylum level).

To a large extent, the dominant species specially determines the ecological and functional structure of the microbial community. Understanding the species composition of a community at various levels can effectively interpret the formation, changes, and ecological impact of the community structure. The species compositions of all samples were counted at the level of classification, phylum, class, order, family, genus, and species. Then, the changes of species abundance of different samples at different classification levels were visually displayed in the form of stack charts. At the phylum level (Figure 3), the top 10 phyla selected for detailed display accounted for more than 95% of all phyla, including *Proteobacteria*, *Bacteroidetes*, *Firmicutes*, *Epsilonbacteraeota*, *Actinobacteria*, *Spirochaetes*, *Verrucomicrobia*, *Planctomycetes*, *Patescibacteria*, and *Cyanobacteria*. Among the four groups of samples, *Proteobacteria* was the dominant phylum, which was consistent with previous studies, accounting for 79.7% (Autumn), 73% (Winter), 80.4% (Spring), 84.5% (Summer). *Bacteroidetes* accounted for 12.5%, 13.1%, 2.2%, 2.5%, *Firmicutes* accounted for 0.05%, 5.4%, 13.9%, 6.9%, successively, and other phyla accounted for less than 6%. 

**Figure 4:**
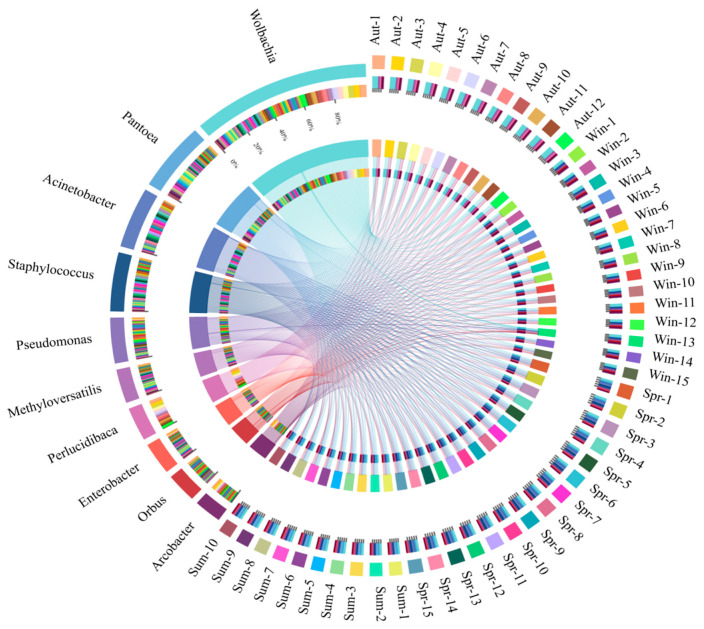
The outermost circle is the name of the samples and the dominant bacteria (at the genus level), and the secondary outer layer represents the bacterial composition and the distribution of relative abundance.

The Figure 4 showed the taxonomic level of the genus, with the top 10 in relative content being *Wolbachia*, *Pantoea*, *Acinetobacter*, *Staphylococcus*, *Pseudomonas*, *Methyloversatilis*, *Perlucidibaca*, *Enterobacter*, *Orbus*, and *Arcobacter. Wolbachia*, as the dominant group, accounted for 31.6%, 14.6%, 14.1%, and 12.6% of the samples in the four groups. Compared with the other three groups, the content of *Wolbachia* in the midgut of adult mosquitoes was most abundant in autumn. *Pantoea* and *Methyloversatilelis* had little change in winter and spring Nevertheless, the content of both of them was the highest in summer and the lowest in autumn. *Acinetobacter* appeared in both spring and summer, with the highest abundance in spring, accounting for 18.4%, and the lowest in autumn and winter. *Staphylococcus* had the highest level of abundance (12.5%) in the group of spring and the lowest in the winter group. The content of *Pseudomonas* in winter was 9.8%, which was significantly higher than the other three groups. *Perlucidibaca*, accounting for approximately 14.5%, had the highest abundance in the autumn. *Enterobacter* and *Orbus* were lower in autumn and winter but were relatively enriched in the spring and summer groups. On the contrary, the level of *Arcobacter* in spring and summer was significantly lower than that in autumn and winter.

### 3.2. Diversity Analysis

**Figure 5:**
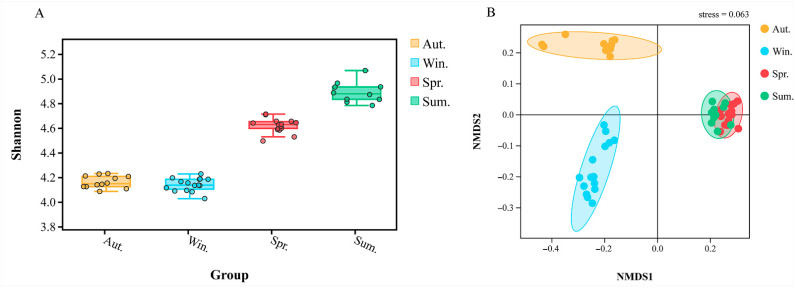
The box plot shows the alpha diversity of different groups, the abscissa is the status of each group (different colors), and the ordinate is the size of the corresponding alpha diversity index (**A**). The beta diversity index between groups was showed by NMDS, stress = 0.063 (**B**).

The differences in microbial alpha diversity could be caused by driving factors in a different environment. The box plot (Figure 5A), combined with grouping information and Shannon index, was drawn by using the test results of Tukey HDS. Therefore, we can find that the α diversity index from high to low was: winter, autumn, spring, and summer. On the whole, the four groups of samples had a low degree of dispersion and high stability. 

**Figure 6:**
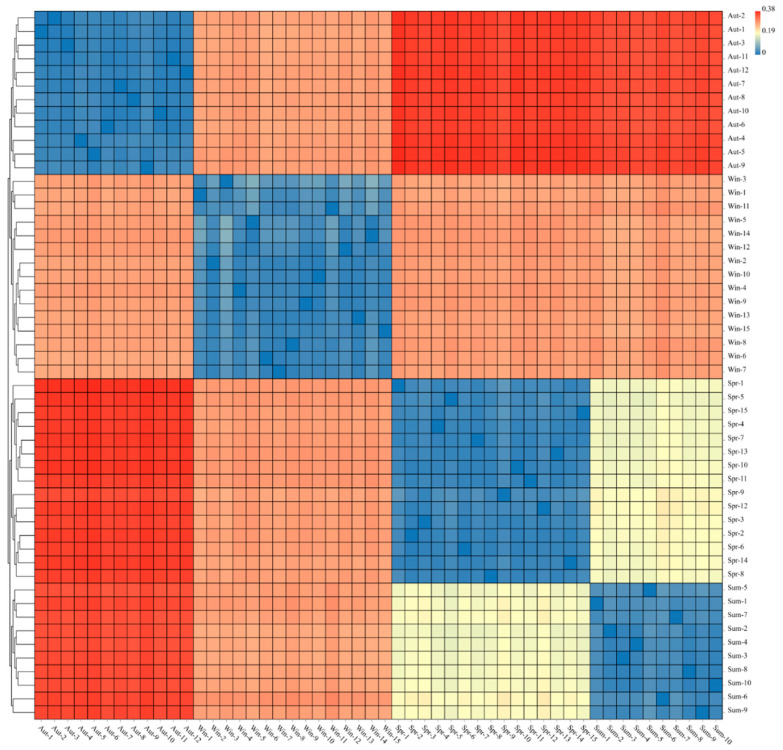
The heat map is used to display the sample distance, the rows and columns are the samples, and the color represents the difference in distance between samples.

In the study of microbial β diversity, Weighted Unifra is one of the common methods used for calculating sample distance. The evolutionary relationship (base variation information) between OTUs was considered plenty, as well as the change of species availability and species abundance, to better evaluate the actual biological significance of diversity of the microbial community. The heatmap was used to analyze and merge the samples with smaller distances into the same cluster. As shown in the Figure 6 above, we can know that according to the different seasons, the bacterial community structure of each sample can be clustered with great reproducibility. The color comparison showed that there was a high similarity in the community composition between the groups of autumn and winter, while the difference between spring and summer was not notable.

Based on the distance index between samples, the Vegan package of R language was used for NMDS analysis. At the genus level, NMDS reduction analysis relied on distance ranking and was used to better study the relationship of bacterial community structure between groups. The model proved to be reliable and accurate due to the NMDS stress = 0.063 (stress < 0.1). Figure 5B indicated that the bacterial community structure of spring was most similar to the group of summer; however, there were significantly different bacterial communities between spring and summer, which was consistent with the heat map.

### 3.3. Differential Species Analysis

**Figure 7:**
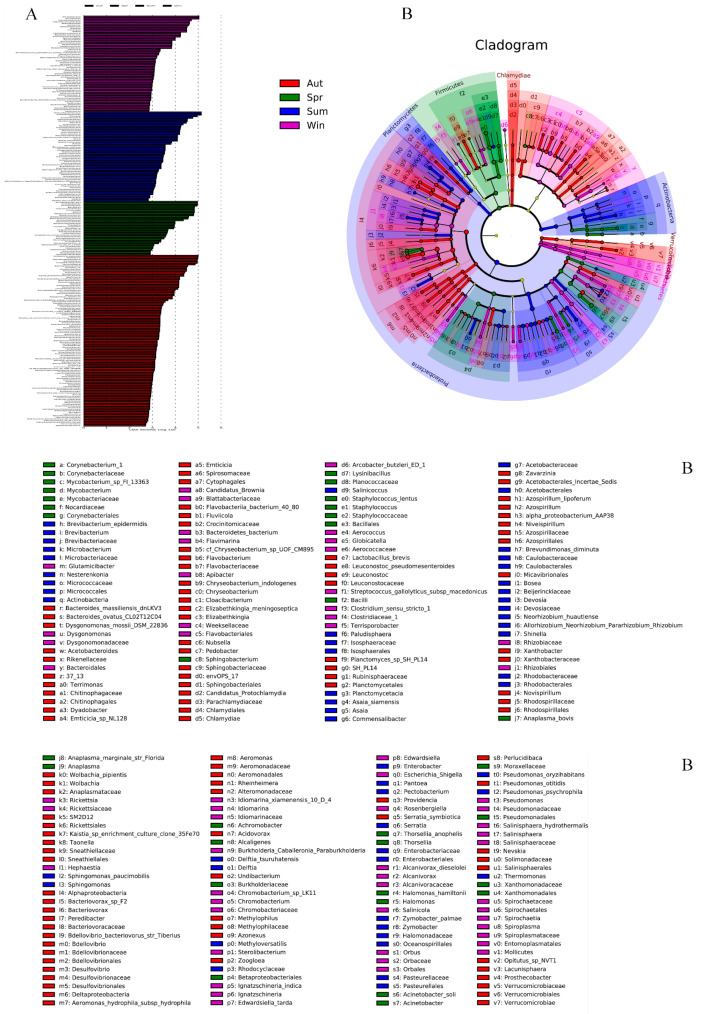
The LEFse chart analyses of all species between groups and outputs a LDA diagram (**A**), the length represents the influence of different species (LDA Score). In the evolutionary cladistic diagram (**B**), each circle represents a specie at different level.

LEFse firstly conducted Kruskal—Wallis test (a test method commonly used for comparison of multiple samples) among all groups of samples, and then used Wilcoxon test used for comparison of two-sample groups to screen out species with significant differences in abundance in different groups. Figure 7A was obtained by using LDA (LDA > 2), which showed the species with significant differences in abundance in different groups. The length of the column represented the size of the influence capacity of different species that was the so-called LDA score. In the cladistic diagram (Figure 7B), circles radiating from the inside to the outside represented the level of classification from the phylum to the genus or species. The diameter of circles was proportional to the relative abundance. Species with no significant differences were uniformly colored yellow. In detail, compared with the other three groups, the abundance of *Bacteroidetes*, *Epsilonbacteraeota*, and *Verrucomicrobia* was rich evidently in autumn at the phylum level. In the group of winter, the abundance of *Spirochaetes*, *Patescibacteria,* and *Cyanobacteria* was higher than the others. In the spring group, the abundance of Firmicutes was significantly increased, and in the summer group, the abundance of Actinobacteria and Planctomycetes was higher than in other groups. Firmicutes enriched distinctly in the group of spring. At the same time, Actinobacteria and Planctomycetes in the group of summer were higher in abundance than others. *Wolbachia, Perlucidibaca, Arcobacter,* and *Bacteroides* played a vital role in the group of autumn at the genus level. *Pseudomonas, Orbus*, *Arcobacter,* and *Streptococcus* existed as differential species in the samples of winter. In the spring group, *Acinetobacter*, *Thorsellia*, and *Staphylococcus* had higher levels of abundance. *Pantoea, Methyloversatilis*, and *Asaia* had significant differences in abundance in the summer.

**Figure 8:**
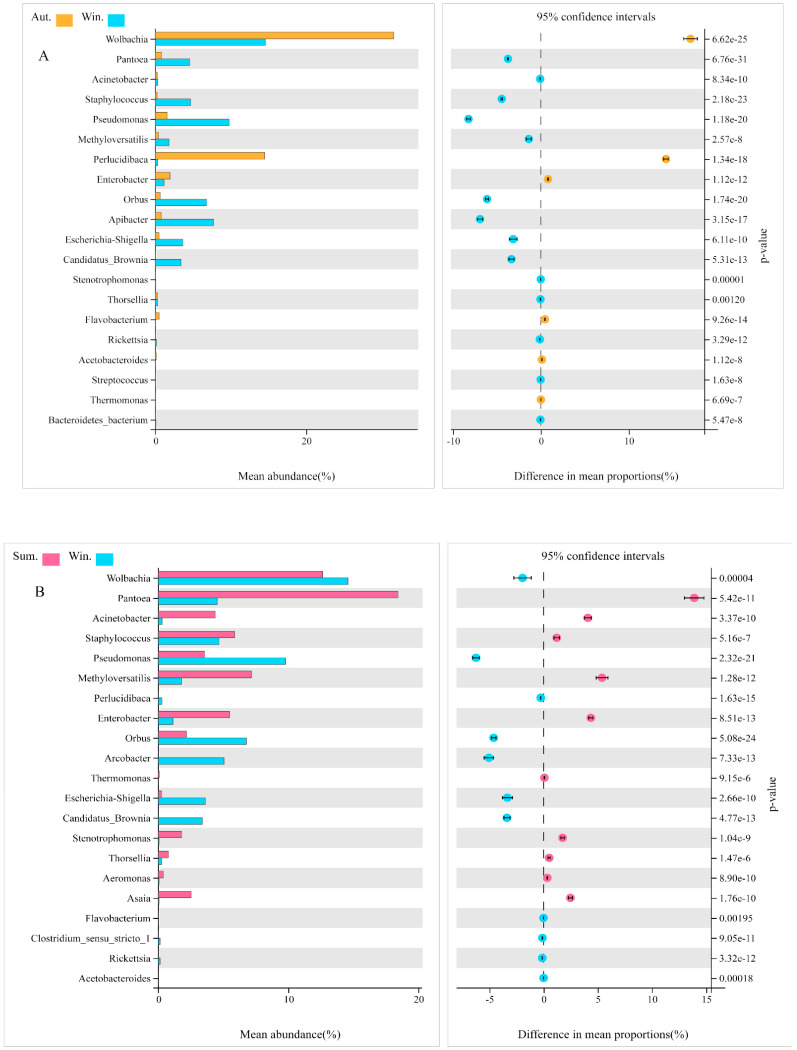
The differentially expressed bacteria between groups screened by Welch’s test. Comparative analysis of the relative abundance of species between autumn and winter (**A**). Comparative analysis of the relative abundance of species between summer and winter (**B**). The display is screened according to *p* < 0.05.

In order to explore the changes in the microbe’s community in the midgut of mosquitoes in the seasons of autumn and winter, we had compared the groups of autumn and winter and the groups of summer and winter at the genus level in particular. The abundance of *Wolbachia, Perlucidibaca, Enterobacter*, *Flavobacterium*, *Thermomonas,* and *Acetobacteroides* was enriched in autumn significantly, compared with the group of winter (Figure 8A). On the contrary, in the group of winter, *Pantoea, Acinetobacter*, *Staphylococcus, Pseudomonas, Thorsellia*, and *Richettsia* were higher than that in autumn, remarkably (*p* < 0.05). In the comparison between summer and winter, we found that the abundance of *Wolbachia*, *Perlucidibaca*, *Pseudomonas*, *Acetobacteroides*, and *Richettsia* in the winter group prominently enriched compared with that in summer (Figure 8B). In the summer group, however, the abundance of bacteria became more active as ambient temperatures rose, among which *Pantoea*, *Acinetobacter*, *Staphylococcus*, *Methyloversatilis*, *Enterobacter*, *Thermomonas*, *Thorsellia*, and *Asaia* had significant differences in abundance (*p* < 0.05).

## 4. Discussion

Generally speaking, the system of the gut is the “second genome” of insects, and the main place for insects to store, digest, and excrete food [1]. The composition of the gut microorganisms of mosquitoes can be varied by species, growth stage, and geographic area [42]. The geographical environment, which determines the source of food and water of the mosquito, is one of the main reasons for the differences in the composition of the microbes of the midgut [43]. In this study, the microbiota of *Cx. quinquefasciatus*, in the tropical areas of Haikou, Hainan, China in the four seasons still maintained high consistency on the whole. According to the analysis of the differences in each season, although the nutritional requirements of *Cx. quinquefasciatus* were usually similar, the relative abundance and diversity of the gut microbiota would still be affected by the living environment [44]. Microbial communities that might be accompanied by changes in the utilization and synthesis of nutrients will have a profound impact on the physiological response of the host.

From the results of the sequencing, Proteobacteria accounted for the largest proportion of the four groups of samples at the phylum level, which is a dominant group with a content of more than 72%. Firmicutes were found in the midgut of *Cx. quinquefasciatus* [45]. The results were consistent with previously reported findings. Firmicutes, as one of the significant microbial species in the gut of insects, played a vital role in the degradation of fiber and cellulose [46]. In addition, Bacteroidetes, which have been found to have the function of degrading high-molecular organic matter [47], could obtain energy and carbon from the degradation of complex carbohydrates [48] and be essential for digestion and fermentation of cellulose [49]. The content of Bacteroidetes in *Cx. quinquefasciatus* in autumn and winter was higher than that in spring and summer. The most likely explanation is due to the drop in environmental temperature, which led to weaker activity of mosquitos and difficulty in feeding. The increasing content of Bacteroidetes was conducive to making full use of its own carbon source to obtain energy to continue life. The most surprising is that *Epsilonbacteraeota*, a type of chemoautotrophic microbe, was first found in the gut of *Cx. quinquefasciatus* in the groups of autumn and winter when the temperature drops. It was reported that this bacterium can oxidize some simple inorganic substances and use chemical energy to reduce carbon dioxide and generate carbon compounds [50,51]. The genome of the bacterium also has the metabolic potential of glycolysis and non-oxidative pentose phosphate pathway, indicating that these microorganisms can synthesize nucleotides and various amino acids necessary for life with the potential ability to degrade polysaccharides, glycosaminoglycans, and polycyclic aromatic hydrocarbons (PAHs) [52]. Nonetheless, the type of this microbe has rarely been studied in depth in the midgut. Epsilonbacteraeota in *Cx. quinquefasciatus* in autumn and winter may be explained by the fact that the mosquitoes were in a dormant state in low-temperature conditions. In order to maintain the requirement for nutrients in the host body, the bacteria were likely to provide carbon sources and other nutrients to the mosquitoes through their unique anabolic functions. *Actinobacteria*, *Spirochaetes*, and *Verrucomicrobia* were also caught in the guts of insects [53,54]. Although only a small proportion in the midgut was occupied by the bacterium of *Spirochaetes* and *Verrucomicrobia* and we know very little about the functions they play in the midgut, some studies still provide references for us. On the one hand, polymers such as pectin and arabinogalactan can be degraded by Spirochaetes. Furthermore, Spirochaetes, which can reduce H_2_ and CO_2_ to produce acetic acid in the midgut [55,56], provided the material basis for the respiration and nutrition of termites [57]. Thus, it was also called acetic acid bacteria [58]. On the other hand, Verrucomicrobia has been found the function of methane oxidation and polysaccharide degradation in the ecosystem [59]. Some members of *Verrucomicrobia* have polysaccharide hydrolase activity, which may assist to degrade cellulose and provide a nitrogen source through their own nitrogen fixation for termites [60,61,62]. At the genus level, *Wolbachia* was the dominant group in the four groups, especially in autumn (the transition from heat to clod). Previous studies demonstrated that *Wolbachia* was naturally present in about 65% of the insects. In the long-term co-evolution process, insects and their symbiotic microorganisms have formed a very close mutually beneficial relationship [63]. *Wolbachia* was called a reproductive microbe because of its ability to affect the fertility of insects [64,65,66]. However, the latest discovery revealed that females occupy a very high proportion of their offspring once insects were infected with *Wolbachia*, particularly in a high-temperature environment [67]. Obviously, a new enlightenment could be control of the population of mosquitoes by way of infection with *Wolbachia* outdoors. The more important finding was that *Aedes aegypti* and *Cx. quinquefasciatus* have significantly enhanced the ability to resist RNA viruses when *Wolbachia* was present [68,69,70]. In addition, the survival rate of drosophila infected by *Wolbachia* raised significantly [71,72]. Scientists argued that might be due to *Wolbachia* having an inhibitory effect on vector-borne viral infectious diseases such as West Nile Virus and Dengue. In this work, the content of *Wolbachia* was higher in autumn and winter, which might be related to the enhancement of the resistance of *Cx. quinquefasciatus* to live. *Pantoea* had also been shown to regulate host behavior: guaiacol could be synthesized by *Pantoea agglomerans* in the gut of desert locusts, leading to the aggregation of desert locusts and causing devastating damage to crops [73]. In female *Anopheles*, Plasmodium berghei can be effectively inhibited by *Pantoea agglomerans,* which can manipulate some immune genes to cut down the proportion of plasmodium carried by 84% to greatly hinder the spread of malaria [74]. Subsequently, *Acinetobacter baumannii* and *Acinetobacter johnsonii* were also isolated from the guts of *Aedes albopictus*, and both of them have been confirmed to assist insects to enhance not only the digestion and absorption of nectar but also blood in the host [75]. The relative abundance of *Pantoea* and *Acinetobacter* was low in autumn and winter, which also confirmed that *Cx. quinquefasciatus* reduced the ability to cope with changes of environment and tried their best over winter. However, the increasing microbial content in spring and summer might lead to significantly enhanced capacity of activity and digestion of blood. The pederin could be generated by *Pseudomonas* in paederus, which can effectively reduce the probability of predation by the natural enemy, so as to protect paderus to a certain extent [76,77]. Potentially, this phenomenon might be in connection with the higher content of *Pseudomonas* in mosquitoes of the winter group, that is, there is an expansion of the food range during food shortages and environmentally unfriendly conditions. *Pseudomonas* has been corroborated that played a role in the metabolism of organophosphates and pyrethroids [78,79]. High levels of *Enterobacter* could produce hemolytic enzymes to promote the digestion of food after blood-sucking [80]. On the nutrient-deficient medium with low-salt and organophosphorus pesticides, the *Enterobacter cancerogenus* in the midgut of *Cotesia vestalis* could grow normally, demonstrating that organophosphorus pesticides could be degraded and eventually transformed into acephate [81]. That might be consistent with the results of our work. In the groups of spring and summer, the content of *Enterobacter* was significantly higher than that in the autumn and winter groups. The reason for this is due to the fact that as the temperature rises with changing seasons, the behavior and metabolism of the mosquitoes were actively vigorous, and the capacity to digest blood, detoxify, and become resistance to pesticides were also enhanced affirmatively. Apart from this, *Asaia* can synthesize vitamin B to provide nutrition for mosquitoes [82,83].

In this study, 16S rDNA sequencing was used to identify the midgut microbiota of *Cx. quinquefasciatus* in the four seasons of Hainan, and we subsequently discussed the linkage between microbes and environmental factors and insecticide resistance. A new idea could be provided for the biological prevention and control of a variety of mosquito-borne infectious diseases, that is, to take advantage of the special functions of the symbiotic microbes to undergo genetic modification to achieve the specific gene or phenotype in the host population. With the rapid development of urbanization, more artificial habitats have been provided for overwintering mosquitoes. Hainan Island has a tropical monsoon oceanic climate. Although we used spring, summer, fall, and winter in sampling time, the four seasons are not distinct. There is also a dry winter, a rainy spring season, and a rainy summer and fall. The research on the midgut microorganisms of mosquitoes in the four seasons, especially overwintering mosquitoes, has been beneficial to enhance the ability of biological control of pests. The density of mosquitoes in early spring and even in summer were directly determined by the number of overwintering mosquitoes regularly. Increasing mosquito density provided the necessary conditions for the widespread of mosquito-borne diseases. There is much sufficiently practical significance to reduce the density of *Cx. quinquefasciatus* in autumn and winter when their activities are weakened, which is of absolute benefit to human beings and the natural environment. To summarize, the microbes of the midgut are often interconnected, and do not act alone. Therefore, in future research, clarifying the interaction between symbiotic bacteria and their functions should also be a top priority.

## Data Availability

The data supporting the findings of this article are included within the article and its Supplementary Materials.

## Supplementary Materials

The following supporting information can be downloaded at: https://www.mdpi.com/article/10.3390/biology11081166/s1. Table S1: Sampling location and seasonal mean temperature; Table S2: Monitoring number of mosquito species; and Table S3: Sequencing data and quality statistics.
