# Supplementary material for: Seasonal Variation of Midgut Bacterial Diversity in Culex quinquefasciatus Populations in Haikou City, Hainan Province, China
Source: Biology (Basel). 2022 Aug 3;11(8):1166. doi: 10.3390/biology11081166 (PMC9405131; doi:10.3390/biology11081166)
Supplement: Supplementary file 1 [file biology-11-01166-s001.zip › biology-1815936-supplementary.pdf]

Supplementary Information

Seasonal Variation of Midgut Bacterial Diversity in *Culex quinquefasciatus* Populations in Haikou City, Hainan Province, China

Penghui Suo, Kaixuan Wang, Hongxiao Yu, Xiuhao Fu, Liping An, Biswajit Bhowmick, Jiachao Zhang and Qian Han

Table S1 Sampling location and seasonal mean temperature

	Autumn	Winter	Spring	Summer
110.34125,20.059658	25.9℃	19.7℃	26.3℃	29.3℃
110.349765,20.066619	25.9℃	19.8℃	26.4℃	29.4℃
110.335321,20.062502	25.9℃	19.7℃	26.2℃	29.3℃

Table S2 Monitoring number of mosquito species

Date	Number
2021.09.10	561
2021.09.21	445
2021.09.30	416
2021.10.09	420
2021.10.20	352
2021.10.31	637
2021.11.10	620
2021.11.21	562
2021.11.30	771
2021.12.11	181
2021.12.22	198
2021.12.30	217
2022.01.09	290
2022.01.19	202
2022.01.31	248
2022.02.09	229
2022.02.18	238
2022.02.28	234
2022.03.10	229
2022.03.21	340
2022.03.30	328
2022.04.09	305
2022.04.20	366
2022.04.29	343
2022.05.10	458
2022.05.21	538
2022.05.30	636
2022.06.11	542
2022.06.22	571
2022.06.30	469
2022.07.10	682
2022.07.21	775
2022.07.31	810
2022.08.11	749
2022.08.21	654
2022.08.31	879

Table S3. Sequencing data and quality statistics.

Samples	Raw Tags	Clean Tags	Effective Tags	Effective Ratio (%)	Max length	Min length	N50
Aut-1	129182	128746	108276	82.38	473	202	461
Aut-2	133156	132594	111281	82.03	474	204	461
Aut-3	134496	133959	112240	82.01	474	213	461
Aut-4	125747	125323	105067	82.18	473	217	461
Aut-5	118953	118496	100299	82.9	472	205	461
Aut-6	133674	133154	114563	84.2	473	206	461
Aut-7	128140	127585	110155	84.35	473	202	461
Aut-8	121308	120772	104115	84.24	474	206	461
Aut-9	125103	124509	109014	85.63	473	206	461
Aut-10	131252	130742	111836	83.71	474	213	461
Aut-11	124324	123787	105423	83.16	472	210	461
Aut-12	128169	127640	107934	82.63	471	223	461
Win-1	125976	125324	113108	87.87	470	210	461
Win-2	117857	117204	104744	87.11	472	201	461
Win-3	130472	129806	119174	89.46	474	206	461
Win-4	121588	121101	107545	86.74	471	206	462
Win-5	128890	128242	111970	85.05	472	225	462
Win-6	121981	121425	108225	87.05	474	213	461
Win-7	119529	118912	107074	87.81	473	205	461
Win-8	126144	125503	111553	86.69	472	205	461
Win-9	132015	131420	116710	86.67	474	201	461
Win-10	123375	122705	108787	86.41	470	218	461
Win-11	130669	130029	118209	88.65	470	213	461
Win-12	125223	124694	110570	86.53	472	204	462
Win-13	133448	132783	118939	87.18	474	214	461
Win-14	127533	126991	110656	84.93	472	214	462
Win-15	128653	128170	114714	87.59	473	223	463
Spr-1	132533	132089	119783	88.83	473	205	466
Spr-2	127994	127527	117902	90.52	474	218	466
Spr-3	120432	119945	108410	88.41	473	218	466
Spr-4	132403	132034	123256	91.59	473	210	466
Spr-5	117903	117579	112113	93.4	470	224	466
Spr-6	131465	131162	121718	91.29	473	225	466
Spr-7	118341	118002	109574	91.11	473	210	466
Spr-8	130625	130173	119734	90.19	474	218	466
Spr-9	119462	119108	109973	90.65	473	220	466
Spr-10	121017	120614	110974	90.1	474	205	466
Spr-11	132778	132386	122463	90.62	473	210	466

Spr-12	124319	123943	110875	87.65	470	218	466
Spr-13	133638	133217	122187	89.9	474	230	466
Spr-14	135710	135203	122339	88.69	473	202	466
Spr-15	130647	130128	121559	91.42	470	218	466
Sum-1	134059	133637	128788	94.57	472	209	466
Sum-2	125635	125049	111233	86.96	474	205	466
Sum-3	131790	131318	120908	90.26	474	206	466
Sum-4	133932	133284	121428	89.08	474	208	466
Sum-5	118314	117820	112657	93.58	473	219	466
Sum-6	122328	121774	107087	86.06	473	205	466
Sum-7	122665	122313	118076	94.63	473	209	466
Sum-8	127303	126719	116525	89.98	469	218	466
Sum-9	129747	129198	118142	89.52	473	210	466
Sum-10	128803	128164	116510	88.98	474	202	466
